# Notes and News

**Published:** 1902-09-20

**Authors:** 


### Notes and News.

The Royal Commission on Physical Training in Scotland is sitting in Edinburgh.

Last week there were eleven cases of plague in Mauritius, one of which was fatal.

The Sanitary Congress closed with discussions on tuberculosis and matters relative to sewage.

There are now 70 cases of small-pox under treatment in London, all at the Long Reach Hospital.

One outcome of the visit of Sir Francis Lovell to the tropics, with a view of creating interest in the study of tropical diseases, has been the donation of a lakh of rupees by Mr. Bomanji Dinshaw Petit to the London School of Tropical Medicine.

Messrs. W. H. Bailey and Son have issued an illustrated catalogue of aseptic hospital furniture which is very complete, and will be found useful by those who have to do with the furnishing of new hospitals, or with that more common task of replacing the fittings of old hospitals with modern and up-to-date aseptic appliances.

Small-pox has made rapid strides in Barbados since the commencement of the outbreak. There have been upwards of 170 cases. All cases and contacts are isolated as far as possible, but the inhabitants have shown themselves somewhat difficult to control. The disease is of a mild character. Some of the buildings erected for the Boers are being requisitioned for isolation purposes.

The use of the Finsen light for lupus has become so popular that a good many hospitals throughout the country have now an installation. This is not as generally known as it might be, and numerous patients apply for treatment at the London Hospital, where the light was first in use, who might well obtain the benefit they need nearer home. There are Finsen lights at the Royal Infirmary, Dublin; Addenbrooke's Hospital, Cambridge; the Royal Infirmary, Manchester; and at the Charing Cross and Westminster hospitals. For the convenience of the public we shall be glad to hear from other hospital authorities having installations.

No fewer than 152 tons of fish have been condemned at Billingsgate Market as unfit for food during the past month.

Three ophthalmic hospitals are to be established in Persia—at Teheran, Ispahan, and Tæuris. Ismael Khan is studying with Dr. Galezowski in Paris for the purpose of acquiring information in regard to the organisation of the hospitals.

Messrs. Allen and Hanburys have arranged, for the use of practitioners, a few simple directions for carrying out the open-air treatment of consumption at home. To these a few recipes are added, among which figure several ways of using the various dietetic and other preparations produced by this firm.

Professor Delepine has reported the results of his examination of the food suspected to have caused the outbreak of ptomaine poisoning at Derby. His conclusions are not very reassuring, as he points to the fact that such poisoning may be quite accidental, and as far as one can see resulting from circumstances from which the makers of pork-pies or sausages could not easily guard against. Apparently the materials used were above suspicion. Infection takes place in an unaccountable manner during their preparation.

One of the disadvantages inseparably connected with the right of the profession to be directly represented on the General Medical Council has been well illustrated by the events of the past fortnight. Without, so far as we can see, the slightest valid reason, Mr. Joseph Smith, of Chiswick, has chosen to come forward as a candidate in opposition to Sir Victor Horsley, thus demonstrating the fact that under present conditions it is hopeless to expect any member of the Council to obtain an uncontested re-election, whatever his position and however eminent his services.

We hope that those Coronation committees who have not yet disposed of their surplus funds will give consideration to the claims of the hospitals in their midst, and in this they may well emulate the example set them by Torquay. There the Mayor (Mr. J. F. Rockbey) set before the inhabitants the idea of raising £1,000, with the understanding that the event should be memorialised by devoting the balance towards the endowment of a bed in the Torbay Hospital, and a cot in the Rosehill Hospital for Children at St. Marychurch. The response was admirable, over £1,300 being raised, and about £42 was set aside for the hospitals. On the original date fixed for the Coronation the festivities that took place reduced the balance to £769. After balancing the accounts, there remained £202 for the Torbay Hospital and £209 for the Rosehill Infirmary. The committee of the latter institution will find the contribution especially timely in view of the fact that they have decided not to rebuild their hospital, but to convert "Goldielea," a commodious mansion in the Lower Warberry Road, so as to meet their requirements. Mr. Ball, the Mayor, has made his youngest grandson a life governor of Torbay Hospital by contributing 20 guineas to its funds. He is a life governor himself, and made his three sons the same early in life, so that he has in this way given 100 guineas to the funds.

